# Herpes simplex virus-1 infects the olfactory bulb shortly following ocular infection and exhibits a long-term inflammatory profile in the form of effector and HSV-1-specific T cells

**DOI:** 10.1186/s12974-017-0903-9

**Published:** 2017-06-23

**Authors:** Chandra M. Menendez, Daniel J. J. Carr

**Affiliations:** 1 Departments of Microbiology, Immunology, Oklahoma City, OK USA; 2 Department of Ophthalmology, University of Oklahoma Health Sciences Center, DMEI #415A, 608 Stanton L. Young Blvd, Oklahoma City, OK 73104 USA

**Keywords:** HSV-1, Dissemination, Olfactory bulb, Long-term inflammation

## Abstract

**Background:**

Herpes simplex virus 1 (HSV-1) infection can result in a life-threatening condition known as herpes simplex encephalitis (HSE). Trafficking patterns by which the virus reaches the central nervous system (CNS) following ocular infection are unresolved. We evaluated early viral dissemination pathways following ocular infection that involve trafficking to the olfactory bulb (OB). Additionally, we have characterized the capacity of HSV-1 to establish latency within OB tissue and profiled the local T lymphocyte response over the course of the acute infection into latency.

**Methods:**

Scarified corneas of C57BL/6 or reporter-inducible Rosa mice (Rosa^Td/Tm^) were inoculated with HSV-1 and assessed for viral dissemination into the peripheral nervous system (PNS) and CNS by RT-PCR and confocal microscopy. T cells and the resident microglia activation signatures were analyzed by flow cytometry. T cell effector function in the form of IFN-γ secretion was measured by T cells isolated from OB in comparison to T cells from other nervous system sites known to harbor HSV-1-specific memory T cells.

**Results:**

Following ocular infection, HSV-1 viral titers from nasal secretions were detected as early as 48 h through 8 days post infection (8 DPI). HSV-1 gene expression was expressed as early as 2 days following ocular infection in the OB and was consistent with an enhanced expression in the ophthalmic, maxillary, and mandibular branch of the trigeminal nerve ganglia (TG). Rosa fluorescence protein expression (RFP^+^) representing HSV-1-infected cells from Rosa^Td/Tm^ mice was detected in the OB before other areas of the CNS (2 DPI). Additionally, during acute infection, most infected cells appeared to be anatomically distributed within the OB rather than other regions of the CNS. During latency (i.e., 30 DPI and beyond) despite no detectable infectious virus or lytic gene expression and low levels of latency associated transcripts, total effector (CD44^+^ CD62^−^) CD4^+^ T, CD8^+^ T, HSV-1-specific CD8^+^ T cells, and MHC class II positive resident microglia numbers continued to increase. CD4^+^ and CD8^+^ T cell populations isolated from the OB during latency were capable of responding to PMA/ionomycin in the production of IFN-γ similar to T cells from other tissue that possess latent virus including the TG and brain stem.

**Conclusions:**

It is currently understood that HSV-1 traffics to the TG following ocular infection. We have identified a second conduit by which HSV-1 can directly access the CNS bypassing the brain stem. We have also recognized that the OB is unique in that during HSV-1 latency, latency-associated transcripts levels were marginally above uninfected controls. Despite these findings, the local immune response mimicked the phenotype of an active infection during latency.

## Background

Herpes simplex virus-1 (HSV-1) is a neurotropic pathogen that initially infects mucosa epithelial cells prior to infection of the innervating sensory neurons. It is a very common pathogen with a worldwide seroprevalence range of 50–l90% [1, 2]. To experimentally mimic human infection in animal models, investigators have infected the cornea, nasal mucosa, oral mucosa, or the skin. Depending on the dose, virus strain, strain of mouse, and site of HSV-1 inoculation [3], infection of the CNS can occur and result in an acute and necrotizing disease known as herpes simples encephalitis (HSE). Due to involvement of the frontotemporal region of the brain, HSE is thought to result from infection originating from the trigeminal ganglion (TG, the innervating facial ganglia), the olfactory route during primary infection, or from latent virus in the TG during reactivation [4]. Olfactory receptor neurons (ORNs) are unique in that they are the only peripheral nerve that form direct synaptic contact and terminate within the CNS at the level of the olfactory bulb (OB) [5]. HSV-1 is capable of infecting the CNS via the OB after direct intranasal infection [6–8]. However, there is ambiguity as to when and how HSV-1 infects the OB following ocular infection.

Upon cornea infection of mice, virus invades the sensory nerve by retrograde axonal transport to the neuronal nuclei of TG as early as 2–3 days following infection [9, 10]. Upon resolution of primary infection, active HSV-1 replication is repressed, and the viral genomes are retained in a latent state within the TG [11]. This is accepted to occur by 28 days post infection (DPI) in mice, and latency associated transcripts (LATs) are the predominant viral transcripts expressed [12]. CD8^+^ T cells are known to infiltrate the TG by 5 DPI and facilitate the clearance of active replicating virus [13]. During latency, CD8^+^ effector T cells form direct immunological synaptic contact with latent HSV-1-infected neurons and prevent viral reactivation [14–16]. While HSV-1 latency and memory T-cell mechanisms are well understood to occur in the TG, it is unclear whether virus is capable of establishing latency in other tissues in close proximity to the eye and TG, namely, within the OB. If virus establishes latency in the OB, it could potentially reactivate to cause CNS disease or death of ORNs.

We have found HSV-1 infects the OB simultaneously with the TG. In addition, we recognized that the OB contains a large number of infected cells when examining the tissue immunohistologically during acute infection. However, attempts to recover infectious virus from the OB yielded few infectious virions in comparison to that obtained from the TG or brain stem (BS). This phenomenon appeared to transition into viral latency with few HSV-1-specific LATs detected in the OB. However, HSV-1 memory T cells and activated microglia continued to increase during latency in the OB. This work highlights the OB can act as a portal of entry into the CNS during acute infection and maintain a long-term T cell presence that escalates during latency.

## Methods

### Mice

C57BL/6 J and B6.Cg-Gt(ROSA)26Sor^tm14(CAG-tdTomato)Hze^/J (Rosa^Td/Tm^) mice containing a *loxP*-flanked STOP cassette for the fluorescent protein variant (tdTomato) were obtained from The Jackson Laboratory. HSV gB T-I.1 T-cell receptor transgenic mice (gBT-I.1) were obtained from Dr. Francis Carbone (University of Melbourne, Melbourne, VIC, Australia [17]. Rosa^Td/Tm^ and gBT-I.1 were maintained and mated as homozygous pairs within the Dean McGee Eye Institute vivarium. Males and females were used at age 6–8 weeks. For experimental procedures, all mice were anesthetized and euthanized with a ketamine/xylazine solution followed by cardiac perfusion with 10–15 ml PBS before tissue removal [18]. The use of mice reported herein was approved by the Institutional Animal Care and Use Committee, protocol #16-026-NSI.

### Infection model

Anesthetized C57BL/6 J and gBT-I.1 mice were infected with 10^3^ plaque forming units (PFU)/cornea of HSV-1 (strain McKrae) in 3 μl PBS following corneal scarification utilizing a 25-gauge needle. The concentration of virus was maintained at 10^8^ PFU/ml. Propagation and maintenance of virus was previously described [18]. Rosa^Td/Tm^ mice were infected with 200 PFU of an ICP0-inducible Cre recombinase HSV-1 strain SC16 (HSV-1-Cre) [19, 20].

### RNA isolation and RT-PCR

Tissue was placed in 500 μl TRIzol reagent (Invitrogen) immediately following removal, snap frozen, and stored in a −80°C freezer. For total RNA extraction, tissue was thawed, homogenized, and processed according to the manufacturer’s instructions. RNA pellets were resuspended in 12 to 30 μl of RNA storage solution (Ambion). Up to 1 μg RNA was converted to cDNA using the iScript Supermix (Bio-Rad) and was processed for RT-PCR using the iTaq Universal SYBR green (Bio-Rad) as previously described [21]. All genes were normalized to *Actb* and phosphoglycerate kinase 1 (*Pgk1*) and quantified by the ∆∆C_T_ method. HSV-1-specific genes, *Cxcl10*, *Ifn*γ, and control gene primer sequences have been previously published [21, 22].

### HSV-1 plaque assay

Cornea tear film and exterior nasal cavities were swabbed for shedding HSV-1 with a PBS-soaked Q-tip, mixed in PBS and then titered for plaques on Vero cells (American Type Culture Collection). Tissue was homogenized in 1 ml RPMI 1640 media (Life Technologies) using a tissue-tearor^TM^ (Dremel) on ice, then centrifuged at 10000×*g* for 1.5 min at 4 ° C. The supernatants of serially diluted samples were incubated on Vero cell monolayers for 2 h in 96-well plates and then discarded and replaced with 100 μl media containing 0.5% methylcellulose as originally published [23].

### Immunofluorescence microscopy

Following PBS perfusion, Rosa^Td/Tm^ mice were transcardially perfused with 10 ml of 4% paraformaldehyde (PFA). Whole mouse brains and TGs were removed, immediately placed in 4% PFA, and fixed for 4 h at 4 °C. Brains were subsequently embedded in a 3% agarose/PBS solution and were sectioned with a vibratome (Vibratome 3000 Sectioning System) at 400–500-μm-thick sections. TGs were dehydrated with a sucrose gradient, placed in O.C.T. compound, and were frozen over a dry ice/ethanol slurry. Twenty-five micron sections were generated using a cryostat maintained at 18 °C. Brain and TG sections were then blocked and permeated for 2 h in a 3% BSA and 0.2% Triton X-100/PBS solution. Samples were further stained with the nuclear dye (DAPI) and then washed 3× with PBS. Sections were then mounted on slides with ProLong Gold (Life Technologies) for confocal imaging on an Olympus FluoView confocal laser scanning microscope (Olympus, Center Valley, PA, v5.0).

### Flow cytometry

Following the removal of the olfactory bulb at the indicated time points, tissue was placed in a 2 ml Wheatley Dounce Homogenizer (Fisher Scientific) with 2 ml DMEM media supplemented with high glucose, L-glutamine, and pyruvate (Life Technologies) and 10% FBS. Single cell suspensions were created and processed as previously described [21]. Briefly, 1/10 the sample homogenate was filtered using a 40-μm nylon mesh filter (Fisher), was pre-incubated with 0.8 μg Fc block (CD16/32) (eBioscience) and then was immunolabeled in 1% FBS/BSA. Total T cells were stained for CD45 eFlour 450 (clone 30-F11), CD3e FITC (clone 145-2C11), CD8a PE (clone 53-6.7), and CD4 APC (clone GK1.5) (all eBioscience). Effector (T-EM) and central memory (T-CM) cells were identified by CD45 eFlour 450, CD3e PE-Cy7, CD4 APC-Cy7, CD8a PE, CD44 APC, and CD62L FITC all from eBioscience as described [21]. HSV-1-specific T cells were identified by CD3 eFluor 450, CD8a FITC, and either gB-PE, ICP8-A488, or RRI-A488 tetramers (provided by the NIH tetramer facility) as previously described [21]. All samples were analyzed with the MacsQuant flow cytometer and MacsQuantify software (Miltenyi Biotec).

### Intracellular IFN-γ assay

At 30 DPI, CD8^+^ T cells were isolated from the indicated tissue using a column-based CD8^+^ T cell isolation kit (Miltenyi Biotec, 130-094-973) according to the manufacturer’s instructions. The entire pool of negatively selected (CD8^+^) T cells from the OB, TG, or BS was placed in culture with 1 mL media and was treated with 50.0 ng phorbol 12-myristate 13-acetate (PMA) and 800 ng ionomycin or vehicle control for 3 h as described previously [24]. After 1 h, 0.67 μL GolgiStop protein transport inhibitor solution/monensin (BD Biosciences) was also added to the culture to maintain intracellular IFN-$\gamma$ [24]. Following the assay, cells were immunolabeled for the extracellular markers including CD45 eFlour 450, CD3e PE-Cy7, and CD8-PE. Cells were then fixed with Perm/Wash buffer (BD Bioscience) and were incubated with anti-IFN-γ APC (eBioscience, clone-XMG1.2) before subsequent washing and analysis of cells by flow cytometry.

### Adoptive transfer

HSV-1 transgenic T cells were isolated from the mandibular lymph nodes and spleens of gBT-I.1 mice at 7 DPI. Single cells suspensions were generated and filtered through 40-μM-nylon mesh filters (Fisher). CD8^+^ T cells were purified by utilization of the CD8^+^ T cell isolation kit and then labeled with carboxyfluorescein succinimidyl ester (CFSE) (Sigma). Briefly, cells were labeled in 10 uM CFSE for 15 min and were washed before retro-orbital injection of 1 million cells into latently infected (30 DPI) mice. Twenty-four hours following transfer, OB, TG, and BS tissues were processed for flow cytometry as described above. CSFE-labeled CD8^+^ T cells were identified by CD45 eFlour 450, CD8a PE, HSV-1-specific tetramer directed against gB (KB/HSV-1.SSI)-conjugated to APC and CSFE staining.

### Statistical analysis

Due to normality assessments, non-parametric statistics were performed. Two- tailed Mann-Whitney tests were used for two-group comparisons. The Kruskal-Wallis one-way analysis of variance with a Dunn’s multiple comparison post-test was utilized for multiple-group comparisons. Significant levels were determined with an alpha of *p < 0.05 (95% confidence intervals) and **p < 0.005. All data is graphed as mean ± standard error of the mean (SEM), and each ‘*n*’ value represents tissue from a single mouse. Ocular and nasal secretion measurements were combined so that each *n* value represents cornea or nasal swab titers from one animal.

## Results

### HSV-1 infects the OB as early as the trigeminal nerve following ocular infection

**Fig. 1:**
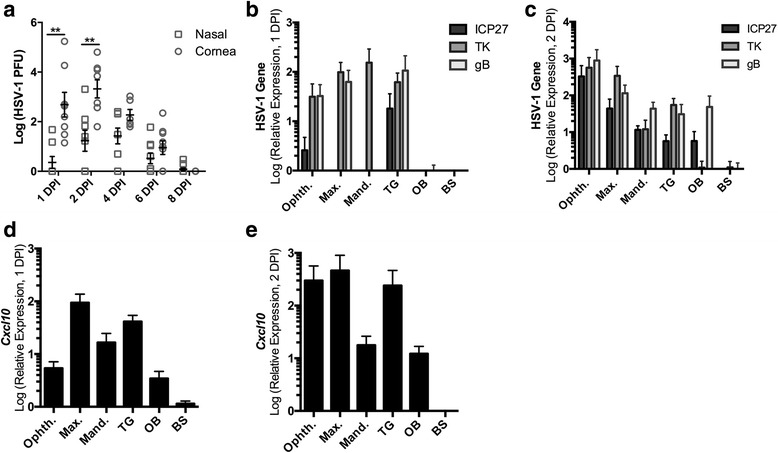
HSV-1 infects the trigeminal branch divisions, TG, and OB early following ocular infection. Mice (*n* = 6–10/group) were infected with 1000 PFU HSV-1/cornea. **a** Swabs were taken from cornea tear film and external nasal cavity secretions and were evaluated for infectious virus by plaque assay from 1 to 8 DPI. **b-c** HSV-1-specific lytic cycle gene expression RT-PCR results from 1–2 DPI (*n* = 3–6/group) of the ophthalmic (*Opth.*), maxillary (*Max.*), and mandibular (*Mand.*) division of the trigeminal nerve ganglia (*TG*), OB, and brain stem (*BS*). **d-e** *Cxcl10* expression measured by RT-PCR at 2 DPI (*n* = 3–6/group). Each *n* value represents sample data from an individual mouse, and 1–2 DPI graphs are represented by mean ± SEM, **p < 0.005 comparing nasal to cornea swabs

Following HSV-1 infection of non-neuronal cells such as cornea epithelium, virus infects the innervating sensory nerve fibers of the trigeminal ganglia (TG) [25]. To determine if HSV-1 is capable of reaching the nasal cavity following ocular infection, the exterior nasal cavities were swabbed for signs of shedding virus in comparison to cornea tear film. Infectious virus was observed in 2 out of 8 mice as early as 1 DPI from the nasal cavity. By 2 DPI, the frequency of HSV-1 detection from the nose increased in which the majority of mice screened (5/8) contained detectable infectious virions (Fig. 1a). However, viral titers were not as abundant as tear samples from the cornea (Fig. 1a). By 4 DPI, cornea and nasal viral shedding were comparable up to 8 DPI, a time point when acute infection is resolved (Fig. 1a). Since virus was detected in nasal swab samples as early as 1 DPI, we next explored a potential pathway of viral dissemination to the nasal cavity from the cornea. The trigeminal nerve contains ophthalmic, maxillary, and mandibular divisions that innervate the cornea, nose, and mouth, respectively [26]. It was hypothesized that virus trafficked to the nasal cavity from the TG by passage of virions from the ophthalmic to the maxillary division. To delineate if virus was capable of reaching the trigeminal nerve divisions innervating the nasal cavity at 1–2 DPI, all trigeminal branches including the nuclei (TG) were analyzed for viral lytic gene expression by RT-PCR. The OB and BS were also included to determine if virus is capable of infecting the CNS at this early time point. Moreover, ORNs innervate the nose, and the BS receives sensory input from the trigeminal nuclei; thus, both could serve as conduits for HSV-1 to disseminate into the CNS [7, 27]. The viral lytic gene transcripts including the (i) immediate-early gene encoding infected cell protein 27 (ICP27), (ii) early gene encoding thymidine kinase (TK), and (iii) late gene encoding glycoprotein (gB) were all detected in the ophthalmic nerve and the sensory root of the TG as early as 1 DPI (Fig. 1b). ICP27 and TK were also identified within the maxillary and mandibular divisions of the TG at this time point (Fig. 1b). All lytic genes measured were expressed at high levels within all trigeminal divisions as early as 2 DPI (Fig. 1c). Strikingly, both ICP27 and TK were observed in the OB by 2 DPI suggesting that the OB (thus CNS) is infected at this early time point, whereas negligible levels were detected in the BS (Fig. 1c).

In C57BL/6 mice following ocular HSV-1 infection, the chemokine CXCL10 is increased in the TG as early as 72 h PI [22, 28, 29]. To determine if HSV-1 gene expression in the trigeminal branch divisions, TG, BS, and OB elicit an inflammatory response, *Cxcl10* was measured by RT-PCR. *Cxcl10* expression was induced in all tissues measured, excluding the BS, as early as 1 DPI with similar levels of expression at 2 DPI (Fig. 1d, e). Thus, inflammation in the form of *Cxcl10* is mounted as early as 1 DPI in the TG, its divisions, and the OB further indicating that infection has occurred at this early time point.

**Fig. 2:**
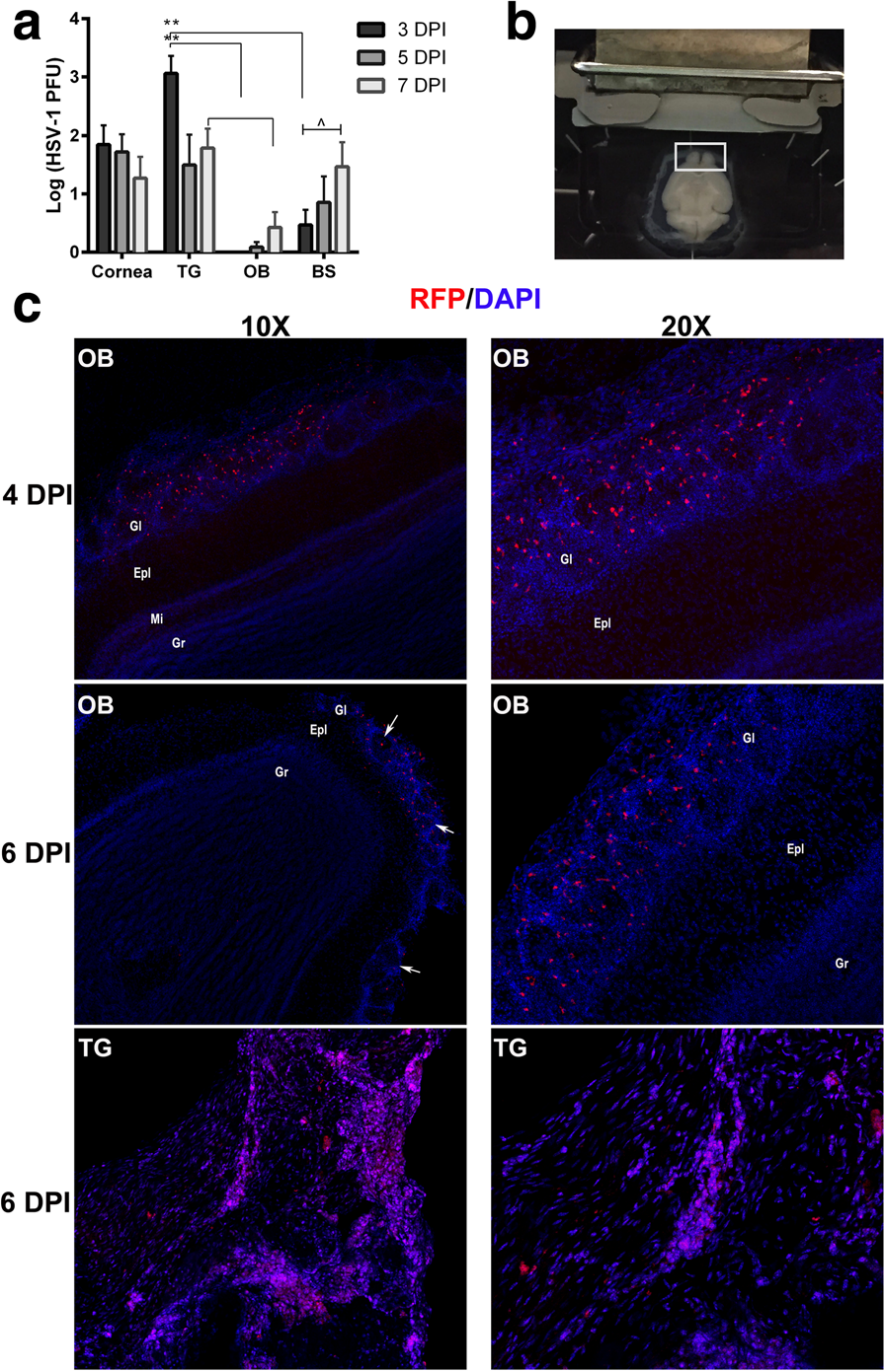
HSV-1 infected cells are observed within the glomerulus of the OB. **a** Infectious virus was measured by plaque assay from the corneas, TG, OB, and BS at 3, 5, and 7 DPI following infection with 1000 PFU HSV-1/cornea of C57BL/6 mice (corneas, *n* = 4–7/group, TG, *n* = 6–9/group, OB *n* = 6–8/group, BS, *n* = 8–12/group). **p < 0.005 was determined by using a Dunn’s multiple comparison’s post-test between tissue at the specified time points and ^*p* < 0.05 comparing 3 and 7 DPI from the same tissue using a Dunn’s multiple comparison post-test following a Kruskal-Wallis one-way analysis of variance. **b**, **c** Rosa^Td/Tm^ mice (*n* = 2/time point) were infected with 200 PFU SC16 ICP0-*Cre*-expressing virus/cornea. **b** Image illustrates transverse sectioning of whole brain tissue for microscopy analysis (white box depicts OB). **c** Representative images of RFP^+^ cells infected with virus localized within the glomerular layer of the OB (Gl) at 4 DPI and 6 DPI. The bottom panels display abundant RFP^+^ labeling within the TG from longitudinally sectioned tissue at 6 DPI. Nuclei are stained with DAPI. White scale bar indicates 40 μM. Other layers of the OB include the external plexiform layer (Epl), mitral cell layer (Mi), and the granule cell layer (Gr)

Since HSV-1 is secreted from the nasal epithelium following ocular infection, it was hypothesized that infectious virus replicates in the OB during acute infection. Plaque assays were performed from 3 to 7 DPI to kinetically monitor HSV-1 dissemination from the cornea (Fig. 2a, b). Infectious virus was recovered from the cornea, TG, and BS from 3 to 7 DPI but was not recovered early (3 DPI) from the OB (Fig. 2a). As time extended following infection, more virus was recovered in the BS consistent with additional virus infiltrating the CNS (Fig. 2a). This occurrence was mirrored in the OB as well, but to a lesser extent (Fig. 2a). To examine HSV-1 localization within the CNS during acute infection, Rosa^Td/Tm^ reporter mouse corneas were infected with *Cre*-expressing HSV-1 recombinant virus (HSV-1 Cre) under the control of the HSV-1-specific ICP0 promoter [22]. Despite modest levels of infectious virus recovered from the OB at 5 DPI (Fig. 2a), HSV-1 infected Rosa^+^ cells were primarily observed within the glomerular level (Gl) of the OB as opposed to other OB regions at 4–6 DPI (Fig. 2c). As a positive control, numerous Rosa^+^ cells and infected tissue were located throughout the TG (Fig. 2c, bottom panel). Surprisingly, there was sparse detection of Rosa^+^ cells in other areas of the CNS in comparison to that observed in the OB during acute infection (data not shown). Collectively, we interpret the data to suggest that the OB provides a unique environment that limits replication and/or the lytic destruction of the cells or neurons in which HSV-1 infects. Instead, virus may be able to more freely traffic through the neurons. Whether this tissue serves as a source of latent or persistent virus infection was addressed in the next section.

### T cells persist and increase in number in the olfactory bulb in the absence of infectious or latent virus during viral latency within the host

**Fig. 3:**
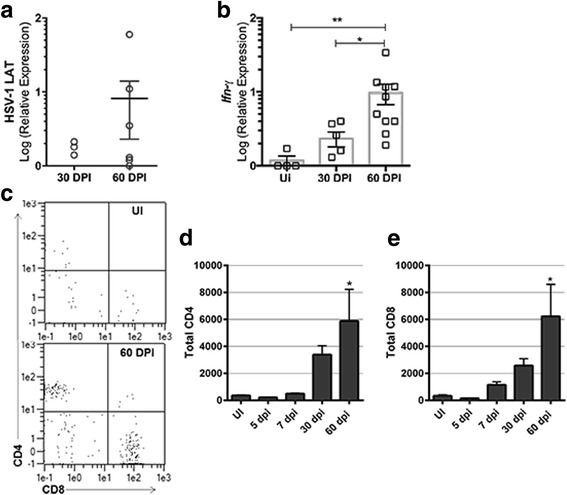
T lymphocyte numbers and *Ifn-γ* expression in the OB increase during latency. The OB was removed and assayed for (**a** LAT or (**b**) *Ifnγ* expression by RT-PCR at 30, (*n* = 6) or 60 (*n* = 10) DPI following infection of C57BL/6 mice with HSV-1 (1000 PFU/cornea). Uninfected (*UI*) samples served as controls. **c** Representative flow plots of CD4^+^ and CD8^+^ T cells (previously gated by CD45^+^ and CD3^+^) from an UI OB sample (*top panel*) in comparison to 60 DPI (*bottom panel*). *p < 0.05 comparing 30 to 60 DPI and **p < 0.005 comparing 60 DPI to UI by applying a Dunn’s multiple comparisons test following the Kruskal-Wallis multiple comparisons test. (**c**–**e**) Total CD45^+^, CD3^+^, CD4^+^, or CD8^+^ T cell counts determined by flow cytometry at the indicated time points (UI-7 DPI *n* = 4, 30–60 DPI, *n* = 5–6). (**c**) Representative flow plot depicting CD4^+^ and CD8^+^ T cells. *p < 0.05 equating 60 DPI to UI (**d**, **e**) also by Dunn’s multiple comparisons post-test

As acute infection is resolved, viral genomes are retained in a latent state, and the production of infectious virions ceases in most tissues [13]. Since the OB was susceptible to infection, and infected cells were retained during the acute phase, it was predicted that OB neurons would become latently infected with HSV-1. LATs were measured by RT-PCR from OB tissue, as an indicator of viral latency during a time period when HSV-1 is known to transition into the latent phase of infection in mice. LAT was detected in samples slightly above background levels (relative expression of 0–1) suggesting a very low level of latent virus present within the OB of mice at 30 DPI (Fig. 3a). LAT expression tended to increase from OB tissue by 60 DPI but was still detected at a modest level insignificantly different to that found 30 DPI (Fig. 3a). Typically, during a latent infection, viral DNA is suppressed in host cell nuclei and forms circular episomes resulting in suppression of active viral RNA expression other than LATs [30, 31]. Since LAT expression was low to undetectable in OB tissue, we reasoned that perhaps lytic gene expression was prominent. However, we were unable to detect any of the immediate early (ICP27), early (TK), or late (gB) lytic gene transcripts from the 30 or 60 DPI OB samples analyzed by RT-PCR (data not shown).

In the TG, certain cytokines such as IFN-γ are expressed upon HSV-1 infection and persist during latency [18, 32]. To determine if an inflammatory response was elicited and maintained in the OB during latency, *Ifnγ* transcripts were measured from total tissue during the latent time periods (30 and 60 DPI). Surprisingly, *Ifnγ* mRNA levels were expressed within the OB and increased from 30 to 60 DPI (Fig. 3b). This led us to examine the tissue for the presence of CD4^+^ and/or CD8^+^ T lymphocytes given that they secrete IFN-γ as an effector molecule upon recognition of HSV-1 infected cells [33]. There was a modest increase in the number of CD4^+^ and CD8^+^ T cells during acute infection that rose significantly during latency (i.e., 60 DPI) (Fig. 3c–e).

### Memory and HSV-1-specific T lymphocytes increase over time in the OB at the onset of latency

**Fig. 4:**
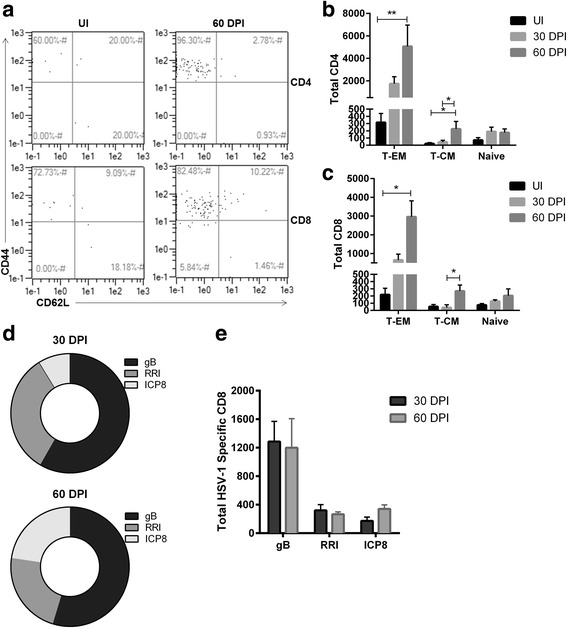
Effector memory, central memory, and HSV-1-specific T cells increase and persist in the OB during latency. **a** Total effector memory (T-EM) and central memory (T-CM) populations were previously gated on CD4^+^ and CD8^+^ T cells (Fig. 3c). Subgates were created to phenotypically characterize T-EM (CD44^+^ CD62L^-^) and T-CM (CD44^+^ CD62L^+^). **a** A representative flow plot for CD4 and CD8 T memory populations is shown from an UI and 60 DPI sample. **b**, **c** Graphs of T-EM versus T-CM populations change from UI (*n* = 4) and 30 to 60 DPI (*n* = 6). *p < 0.05 and **p < 0.005 as determined by Kruskal-Wallis multiple comparisons test between T-EM or T-CM followed by a Dunn’s multiple comparisons post-test between time points. **d** Depiction of the frequency of the CD3^+^, CD8^+^ T cells specific for the HSV-1 immunodominant peptide gB, and subdominant peptides RRI, and ICP8 at 30 and 60 DPI. **e** HSV-1T cell numbers specific for gB represent *n* = 6–7 for each time point, RRI (30 DPI, *n* = 4, 60 DPI, *n* = 10), and ICP8 (30 DPI *n* = 8, 60 DPI, *n* = 4)

Since there was a persistent presence of T cells and *Ifn*γ message in the OB during latency, we next characterized the local T cell active/memory phenotype, which is indicative of previous or persistent antigen exposure. Both CD4^+^ and CD8^+^ T effector memory (T-EM: CD44^+^ CD62L^-^) and central memory (T-CM: CD44^+^ CD62L^+^) cell populations increased substantially during latency with a significant elevation by 60 DPI (Fig. 4a–c). To determine if T cells were HSV-1-specific, CD8^+^ T cells were evaluated for specificity to the immunodominant HSV-1 glycoprotein B (gB_498-505_) and two other subdominant epitopes, infected cell protein-8 (ICP8_171-178_) and ribonucleotide reductase (RRI_822-829_) [34, 35]. Overall, 60% of the CD8^+^ T cells were gB-specific at 30 DPI and remained consistent to 60 DPI (Fig. 4d, e). There were no significant changes in reactivity to CD8 T cell subdominant epitopes RRI and ICP8, although the percentage of RRI-CD8^+^ T cells appeared to equilibrate to ICP8- CD8^+^ (15–35%) levels by 60 DPI (Fig. 4d, e). Total numbers of each HSV-1-specific T cell groups also remained consistent from 30 to 60 DPI with a modest but insignificant rise in ICP8-reactive CD8^+^ T cells (Fig. 4e). These results confirm that a substantial portion of the effector CD8^+^ T cells are likely HSV-1-specific and are maintained within the OB during latency.

We also examined the OB for the presence of T_reg_ cells (CD3^+^, CD4^+^, FoxP3^+^) during latency; however, T_reg_ cell populations were undetectable (data not shown). Thus, in summary, effector memory and HSV-1-specific T cell populations are maintained within the OB during viral latency despite the fact that LAT expression was modest and viral lytic genes were not detected within the tissue during this time period. These results are in contrast to that observed in tissue where viral latency is established, such as the TG. In the TG, viral LATs are abundantly expressed at 60 DPI; however, memory T cell numbers are decreased at 60 DPI compared to 30 DPI levels [21].

### Isolated T cells from the OB exhibit functional adaptive T cell responses during viral latency

**Fig. 5:**
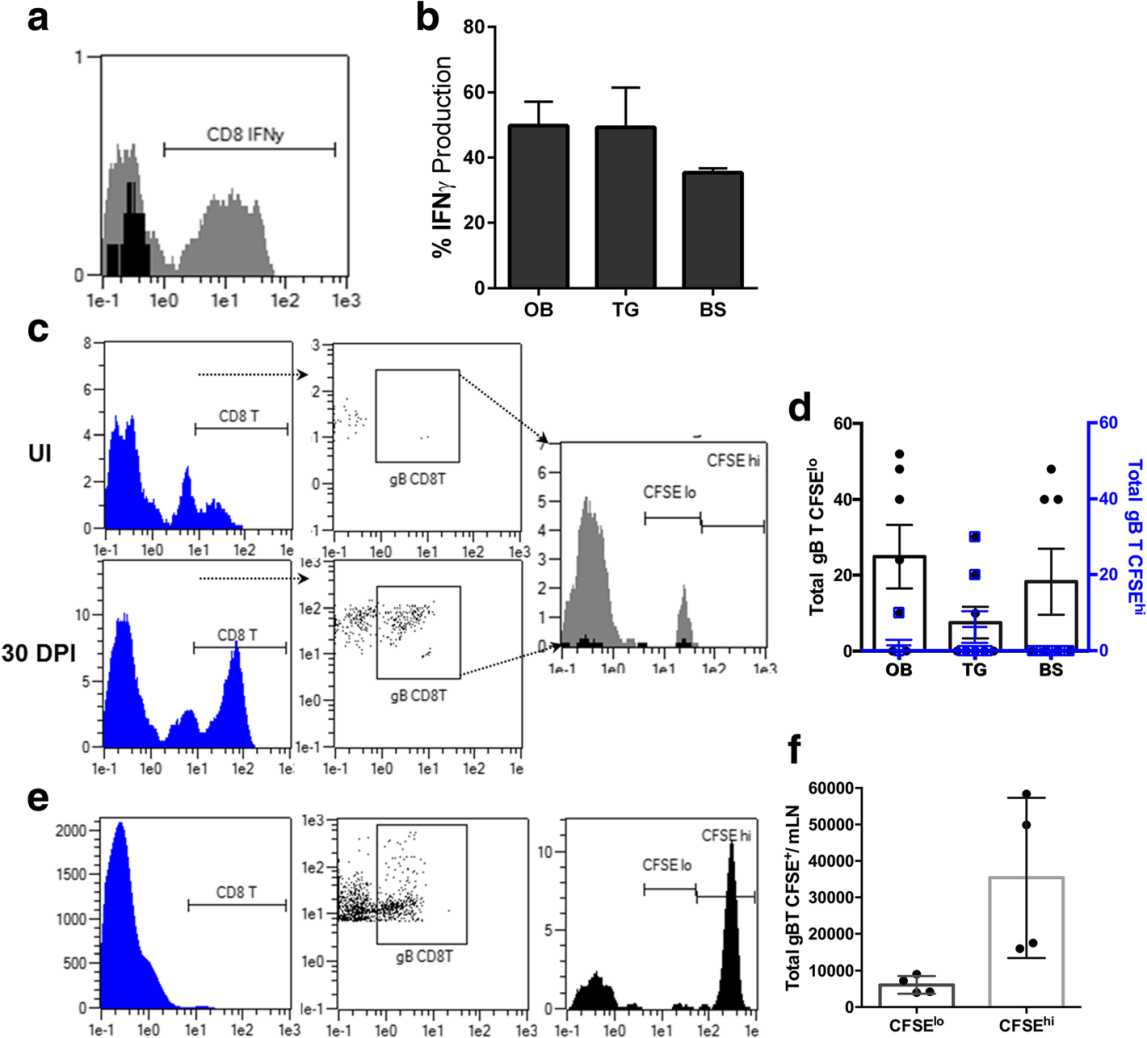
OB T cells exhibit effector function by production of IFN-γ. **a** Histogram representing isolated OB T cells at 30 DPI stimulated with PMA and ionomycin (*grey*) compared to uninfected (*black*). **b** Percentage of isolated CD8^+^ T cells expressing intracellular IFN-γ (OB *n* = 6, TG and BS, *n* = 4). **c** Representation of adoptively transferred HSV-1-specific CD8^+^ T cells gated on CD8^+^ and HSV-1-specific gB-tetramer^+^ and further identified by intracellular fluorescent levels of CFSE from a 30 DPI OB sample (*grey*) compared to UI recipient mouse (*black*). **d** Total number of CFSE^lo^- or CFSE^hi^-labeled HSV-1 gB-specific T cells from the OB, TG, or BS at 30 DPI (*n* = 7–8). **e** Representative plots from adoptively transferred CFSE^+^ gB-specific CD8^+^ T cells within the mandibular LN’s (mLN) at 30 DPI (*n* = 4). **f** Total number of CFSE^+^ gB-specific CD8^+^ T cells within the mandibular LN at 30 DPI

To examine the activation and effector capacity of the resident memory T cell populations present within the OB, OB T cell production of IFN-γ was compared to that of T cells isolated from the TG and BS during latency. At 30 DPI, T cells were removed from the OB, TG, and BS. Intracellular IFN-γ levels were measured by flow cytometry following stimulation with PMA and ionomycin. OB T cells were as capable of responding to the stimulus similar to TG and BS T cells indicating that T cells from the OB are functional effector cells (Fig. 5a, b). Local memory HSV-1-specific T cells are known to self-maintain themselves within the TG [14]. To determine if the population of T cells observed within the OB during latency were proliferating or trafficking from the periphery, transgenic HSV-1 gB-specific T cells were adoptively transferred to infected mice at the time of the establishment of viral latency (30 DPI). When tracking the adoptively transferred HSV-1-specific cells by CSFE, few cells entered the OB, TG, or BS (Fig 5c, d). In contrast, numerous cells were found to home to the draining lymph node (Fig. 5e, f) indicating that cells within the OB, TG, and BS are self-maintained or proliferate.

### Resident microglia numbers and activation increase during latency

**Fig. 6:**
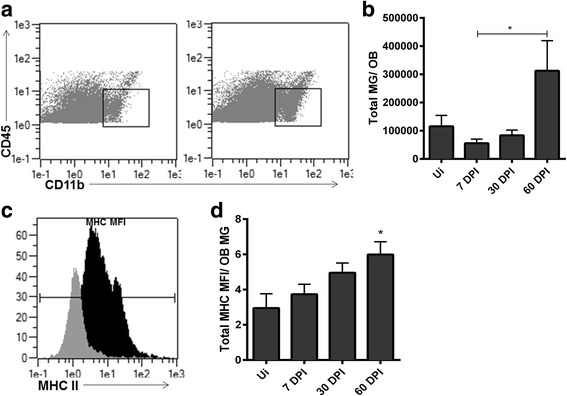
Resident microglia numbers and MHC class II expression on individual microglial cells increase within the OB during latency. **a** Representative flow plot showing CD45^int^, CD11b^+^ cells from OB defined as microglia (*MG*). **b** Total number of MG residing in the OB at times post infection compared to uninfected (*UI*) OB, *p < 0.05 between 7 and 60 DPI by a Dunn’s post comparisons analysis. **c** Representative histogram demonstrating an increase in MG mean fluorescence intensity (MFI) of major histocompatibility complex class II (MHC II) expression comparing 60 DPI (black) to UI (grey) OB MG. **d** Total MG MFI of MHC II expression as determined by flow cytometry, *p* < *0.05 comparing 60 DPI to UI controls with a Dunn’s post-test. For **b** and **d** UI *n* = 4, 7 DPI, *n* = 5, and 30 and 60 DPI, *n* = 7

We previously recognized microglia activation in the ependymal region of mice during HSV-1 latency even though T cells were found to be exhausted [21]. Thus, we evaluated the microglia population in the OB during viral latency. Microglia (MG) numbers remained relatively stable through 30 DPI with a modest but insignificant reduction at 7 DPI (Fig. 6a, b). The loss of MG during acute infection may be the result of the cytolytic effect of HSV-1 on MG infected with the virus. Of note, by 60 DPI, the number of MG residing in the OB had significantly increased threefold compared to earlier time points (Fig. 6b). In addition, the expression of MHC class II (MHC II) on MG continually increased during acute infection into latency such that by 60 DPI, there was a significant elevation in the MHC II mean fluorescence intensity (MFI) compared to uninfected control cells (Fig. 6c, d). We interpret the results to suggest that MG are either proliferating and/or migrating to the OB from other regions of the CNS and may play a role in the maintenance of T cells within the OB.

## Discussion

In this study, we report that infectious virus is shed from the nasal cavity as early as the cornea tear film, and lytic gene expression is found in the ophthalmic branch of the TG 24 h PI. Nasal secretion of HSV-1 at 2 DPI also correlated with early infection of the trigeminal divisions, trigeminal nuclei, and OB simultaneously. We were surprised by the high levels of infectious virus recovered from the nasal cavity early following ocular infection. Although each division of the trigeminal branch was infected, we cannot exclude viral passage from the cornea to the nasal epithelium by self-inoculation of the mice during grooming or scratching, or passage from the nasolacrimal duct [36]. It is important to note that sympathetic and parasympathetic fibers of the TG innervate the lacrimal glands and nasal mucosa, and the ophthalmic division directly innervates the dorsum of the nose; a site also innervated by the maxillary division [27]. Consequently, there is likely overlap in viral dissemination pathways to and from the cornea and nasal mucosa. Regardless, we have demonstrated that HSV-1 is capable of infecting the CNS at the level of the OB early (2 DPI) following ocular infection. However, despite detection of HSV-1 genes in the OB by 2 DPI and identification of infected cells in the glomerulus of the OB at 4 and 6 DPI, the amount of infectious virus recovered from the OB during the acute phase of infection was modest. We reason that the OB may act as a conduit for viral dissemination into the CNS rather than serve as a reservoir for viral self-amplification. Along these lines, LAT expression in the OB was low especially when compared to expression in the TG and other tissues of the CNS during latency [21]. These results are consistent with previous work that report only non-neuronal cells within OB tissue expressed LATs by in situ hybridization during acute and latent infection [37]. We surmise that the behavior of the virus within the OB is unique likely due to the anatomical features of the OB, OB neurons, and/or supporting cells [38].

In the current study, T cell numbers continued to increase in the OB during the latent period of infection despite evidence that the OB harbored latent or even lytically active virus. This occurrence correlated with a significant rise in the number of MG as well as their state of activation. We did not investigate HSV-1 antigen expression by resident MG during latency as there was little LAT and no lytic gene expression. Yet, HSV-1-specific CD8^+^ T cells were maintained in number throughout the course of latency comparing 30 to 60 DPI. Since effector/memory T cells continued to rise in number in the OB during latency and yet, little HSV-1-specific CD8^+^ T cells were recruited to this tissue following adoptive transfer, we conclude the effector/memory T cell population maintained cell numbers independent of circulating populations. This is similar to what has been reported in the TG [14, 34]. The OB is the only nervous tissue that contains cells (i.e., ORNs) that directly contact the external environment while also forming direct neural connections with the CNS. Therefore, it would be interesting to know if the expansion of memory T cells that occurred following HSV-1 infection also occurs following other pathogen insults to determine if the OB provides a unique environment to maintain adaptive memory T cell responses. Additionally, whether the expansion of effector/memory T cells within the OB provides advantageous protection from future HSV-1 infections, or similar pathogen encounters, is currently unexplored.

## Conclusions

The identification that HSV-1 readily infects the OB early following ocular infection sheds light on the complex and multiple pathways the virus utilizes to disseminate to other tissues including the CNS. In addition, the route(s) by which HSV-1 freely traffics to each of the trigeminal pathways and to other mucosa epithelium may be pertinent towards vaccine development or when designing therapeutics for patients who suffer from disease as a result of viral reactivation. Considering OB neurons form connections with integral brain regions, and infection of olfactory neurons enables viral migration to the brain amygdala and hippocampus [39], infection of these tissues could have severe consequences. In fact, there is evidence that HSV-1 DNA resides in 15% of postmortem human OB tissue [40].Therefore, by recognizing a chronic inflammatory response persists in the OB during latency could further provide evidence that HSV-1 infection by the olfactory nerve route could potentially cause neuronal damage. On the other hand, understanding how the OB is unique in harboring a prolonged activated immune cell or memory T cell signature may be advantageous by allowing protection from future encounters of identical or antigenically similar pathogens.

## Abbreviations

(BS): Brain stem
(CFSE): Carboxyfluorescein succinimidyl ester
(DPI): Days post infection
(Epl): External plexiform layer
(GB): Glycoprotein B
(Gl): Glomerular layer
(Gr): Granule cell layer
(HSE): Herpes simplex encephalitis
(HSV-1): Herpes simplex virus-1
(ICP27): Infected cell protein 27
(IFN-γ): Interferon gamma
(LAT): Latency associated transcripts
(Mand.): Mandibular
(Max): Maxillary
(OB): Olfactory bulb
(Opth.): Ophthalmic
(PFA): Paraformaldehyde
(PMA): Phorbol 12-myristate 13-acetate
(RFP): Red fluorescent protein
(T-CM): T central memory cells
(T-EM): T effector memory cells
(TG): Trigeminal ganglia
(TK): Thymidine kinase
